# Under pressure

**DOI:** 10.1038/s44319-025-00419-3

**Published:** 2025-03-18

**Authors:** Bernd Pulverer

**Affiliations:** EMBO, Meyerhofstrasse, Heidelberg, 69117 Germany

**Keywords:** Economics, Law & Politics, Science Policy & Publishing

## Abstract

Academic and federal institutions supporting research and healthcare in the USA face suffocating legislation and severe cutbacks, with dire knock-on effects on global research. How can the international scientific community buffer the impact.

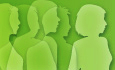

US research has been under political pressure for some time. Examples include regulatory overreach on federal stem-cell research informed more by religion than scientific arguments, a growing vaccine skepticism, or the chilling effects of draconian visa and immigration policies, which dampen the international mobility that has underpinned the dominance of the USA in the biosciences (Elias et al, 2024).

After the US election on Nov 5, many expected that things would get worse—after all, the challenges fired at academia through a relentless barrage of presential executive orders had all been vocalized for years by US President Donald Trump’s supporters and proponents of the populist ‘MAGA movement’. A good example is the speech US Vice President JD Vance gave at the National Conservativism conference on Feb 21, where he labeled universities as “the enemy”. The notion that universities are “fundamentally corrupt” and “pursue deceit and lies”—such as vaccination or climate change—apparently in order to keep the population under control by subterfuge in pursuit of an ill-defined nefarious agenda builds on populist ideas far predating current politics.

The US administration aims to undermine academia under the guise of increased government efficiency—the nebulous mandate of DOGE, executed by unelected, non-expert ‘advisors’ who themselves built economic empires based on the education and research provided by US academic institutions. The assaults on academia and research are multifaceted by design.

Undermining academic institutions: limiting overheads for federal funding to academic institutions to 15% effectively reduces research investment by an estimated US$4 billion. As Mikael Elias and Lynn Kamerlin argue in this issue, the previous NIH overhead/indirect cost rate of, on average, 27% was largely in line with both private-sector spending and overheads awarded by European funding agencies (Elias and Kamerlin, 2025). The effect may be dramatic cutbacks to research infrastructure at US universities or increased bench fees to compensate (the US research budget is still unsettled despite a vote in the US Senate to increase funding).

Undermining federally supported scientific infrastructure and funding: the administration appears to follow a three-fold strategy to thwart academic funding: limiting overheads, a communications embargo at federal institutions to block grant award meetings and by cutting their staff. The proposed cuts and layoffs—many challenged in the courts—at the CDC, NIH and NSF are already causing a state of insecurity among public servants who keep the cogs of the US research infrastructure turning, who distribute the majority of US research funding and who play a decisive role in global-health management (the NIH is the world's largest supporter of medical research). The long-term impact in the USA and beyond is hard to quantify, but contingency plans are urgently needed. In particular, data resources essential for global research, such as CDC’s public-health databases or clinicaltrials.gov and PubMed by the US National Library of Medicine (NLM), may eventually be compromised. Long-running data-series may be irrevocably undermined even by a temporary pause. Alfonso Valencia notes in this issue that “the centralized nature of these resources renders them susceptible to censorship, technical failures, or even manipulation” (Valencia, 2025). He advocates decentralization, citing examples such as the European Genome-Phenome Archive (EGA) and ELIXIR. The jittery performance of both the PubMed and PubMedCentral (PMC) databases on March 2 may serve as a wake-up call to re-discuss the resilience of US-based database services that the global biosciences community depends upon. PMC is mirrored by EuroPMC at EMBL-EBI and served a record number of queries during the outage, but it is ultimately co-dependent on NLM.

Loss of international talent: insecure funding and visa restrictions (Elias et al, 2024) are affecting international applications at a time when postdoc numbers have already been declining as more PhDs chose biotech or other career paths with better job security and compensation. Even senior academics are considering their options in Europe and beyond, as a doubling of applications from the USA for principal investigator positions at Institutes of the Max Planck Society seems to indicate—its president Patrick Cramer did not delay to invite applications from the ‘new talent pool’. Losing international talent would challenge the USA’s dominant position in the biosciences with knock-on effects on biotech and pharmaceutical companies.

Loss of a creative research environment and academic independence: The ‘banning’ of DEIA policies and any articulation of related or even unrelated terminology around inclusion, equity, and diversity at federal institutions, including CDC and NSF, creates a work environment governed by fear of sanctions which is not conducive to innovative research based on open discussion and free thinking. The rejection of grant applications or the (silent) alteration of the published record or database entries on account of the use of medically-relevant terminology such as ‘bias’, ‘gender’, ‘trauma’, ‘ethnicity’ and even ‘female’ is unacceptable. As Jocalyn Clarke and Kamran Abbasi point out that “sex and gender data … are essential for understanding differences in populations and among individuals” (Clarke and Abbasi, 2025). EMBO Press joins other journals like the BMJ in not allowing such changes to the published record or to accepted research papers and in not allowing authorship changes based on the use of such words in a scientific context.

Deregulation and commodification: The current administration appears intent on improving economic performance by deregulation, including the governance of ethically sensitive research. The latter is exemplified by dialing back any restraints on research on AI. While this may yield short-term economic benefits, it ignores the benefits of research for humanity and the planet.

Even if the courts—already overwhelmed with challenges to the barrage of executive orders—block some of these changes, the period of uncertainty while cases percolate up the courts will cause lasting damage by undermining in particular short-term positions and research in highly competitive areas. Furthermore, the reputation of the USA as a reliable partner for international collaboration will suffer. This might be particularly acute for jointly financed and hosted research projects and infrastructures such as PDB (Japan, Europe, USA) or PubMedCentral (USA, Europe).

The USA has been the pacemaker of world affairs since the second world war. Uncoupling this role from global research risks undermining the all-important synchronization of international science networks. More worrisome are the ongoing political and social movements in Europe and elsewhere that mirror the policies by the current US administration. European institutions need to resist the temptation to jump on the bandwagon of deregulation to unleash economic growth. The refusal of the UK to sign the declaration of the AI action summit in Paris in January is a case in point.

Better education and communication of scientific method and fact is required to immunize the public against horse whisperers peddling alt facts by being better informed of the value of both basic and applied research. Take, for example, the recommendation by Robert F. Kennedy Jr., the new health and human services secretary, to use cod liver oil—implicitly instead of MMR vaccination—to fight the ongoing measles outbreaks in the USA. In line with the apparently systematic strategy to turn criticism of the government on their head, he followed, “We’re going to be honest with the American people for the first time in history about what we know, what we don’t know and that’s going to anger some people who want an ideological approach to public health.” The US government needs to be made to understand that frustrating infectious disease control nationally and internationally by defunding WHO and USAID will not only fatally undermine global health, but also US health, with dramatic economic consequences.

Understandably, institutions as much as individuals in the USA have to date mostly been pliant—the silent changes to purge ‘banned terms’ on institutional websites like that of the CDC but also independent institutions like ASM and HHMI, echo Google’s voluntary rebranding of the Gulf of Mexico. It is therefore up to the international community to voice concerns and to hold the line, starting with an explicit exclusion of changes to the scientific literature of terms that affect scientific meaning.

The global scientific community, and the organizations that represent them, should support their US colleagues in articulating the damage these policies will do to the US research environment and consequently the US economy. Pointing out the negative economic impact has the best chance of being heard by the current administration and its supporters. It is crucial to provide evidence that US science is just as much based on international contributions as the rest of the world depends on US contributions to quell the dogma that the USA has been ‘unfairly exploited’ or that other countries steal US prowess to profit unilaterally. It is also essential that the biotech and pharma industry re-affirms the message that private sector R&D relies on academia to underline the fact that part of the public good of academic research translates to economic gain.

The most effective way to counterbalance the attacks on core values of the scientific community is to coordinate responses and send a consistent message to the public and governments here and in the USA. The political turmoil may slow or derail urgent research on public health or climate change—from women’s health issues to ecosystems’ climate adaptation—and impact on our ability to mitigate irreversible damage to global health and the environment. The rest of the world simply cannot stand by while local politics undermine action on global issues.

The close-knit global research network means that a much-reduced contribution of research from the USA would have knock-on effects on many international programs. Global challenges cannot be addressed by nations acting in isolation. Research-intensive nations in Europe and Asia-Pacific need to urgently develop closer cooperation to compensate.

The flurry of changes unleashed by the Trump administration have been dramatic in speed and scope. The intention seems to be to shock opposition into silence and to clog the process of checks and balances through the courts. The plan is as devious as it is effective, as it builds both on fear and on the well-known psychological trait of ‘normalcy bias’ which leads people to disbelieve or minimize threat warnings. Philip Ball poignantly reminded us that academia has before been too easily engaged in ‘anticipatory obedience’. This is easy to diagnose in retrospect, but hard to act on when individual jobs may be on the line and any comment might be avoided for fear of aggravating an already tenuous and hostile situation. While outside voices will matter less to an introverted government, a consistent and constructive argument put forth jointly by major scientific organizations will still be impactful. This should include the message that, precisely because US science capacity and infrastructure is essential for global research and public health, others are willing to host and fund both key infrastructure and talent in lieu of the USA. The implication and the message to the US government is that their divestment of research threatens its current leadership role in the biosciences and its innovative high-tech economy that has been the envy of the world.

Even if the situation is still very much in flux, the time to do so is now before further damage sets in and long-term decline ensues.

## References

[CR1] Clarke J, Abbasi K (2025) Medical journal editors must resist CDC order and anti-gender ideology. BMJ 388:r253 39904526 10.1136/bmj.r253

[CR2] Kamerlin SCL, Elias MH (2025) NIH’s 15% Cap: a cost comparison and research outlook. EMBO Rep 10.1038/s44319-025-00418-4 10.1038/s44319-025-00418-4 PMC11977179 40102587

[CR3] Elias M, Sompiyachoke K, Fernandez FM, Kamerlin SCL (2024) The ineligibility barrier for international researchers in US academia. EMBO Rep 25:457–458 38263328 10.1038/s44319-023-00053-x PMC10897176

[CR4] Valencia A (2025) Decentralized databases in biomedical research: lessons from recent events; EMBO Rep 10.1038/s44319-025-00417-5 10.1038/s44319-025-00417-5 PMC11977236 40102589

